# Ossification of the Pediatric Elbow with a Focus on Radial Head Ossification Patterns

**DOI:** 10.1007/s12178-026-10047-6

**Published:** 2026-06-23

**Authors:** Tina H. Tran, Hannah Chi, Tiffany C. Liu, Bamidele Kammen, Ishaan Swarup

**Affiliations:** 1 Department of Pediatrics, University of California San Francisco Benioff Children’s Hospitals, Oakland, CA USA; 2 Department of Orthopaedic Surgery, University of California San Francisco, San Francisco, CA USA; 3 Department of Orthopaedic Surgery, Longview Orthopedic Associates, Longview, WA USA; 4 Department of Pediatric Radiology, University of California San Francisco Benioff Children’s Hospitals, Oakland, CA USA; 5 Department of Pediatric Orthopaedic Surgery, University of California San Francisco Benioff Children’s Hospitals, Oakland, CA USA; 6 UCSF Benioff Children’s Hospital Oakland - Pediatric Orthopaedics, 744 52nd Street, Oakland, CA 94609 USA

**Keywords:** pediatric elbow, eccentric ossification, radiocapitellar alignment, radiocapitellar line, radial head ossification

## Abstract

**Purpose of Review:**

Accurate interpretation of pediatric elbow imaging depends on understanding the developmental relationship between the radial head and the capitellum. While the traditional sequence of pediatric elbow ossification centers provides a useful framework, it does not capture morphologic variability, asynchronous maturation, and eccentric ossification patterns that may mimic pathology. This review synthesizes current evidence on pediatric elbow ossification with particular emphasis on normal and variant radial head development. The following key questions are addressed: (1) How frequently does eccentric radial head ossification occur on MRI? (2) What is its magnitude and relationship to age and sex? (3) How should normal variants be distinguished from pathology?

**Recent Findings:**

Recent findings reveal that the capitellum commonly ossifies eccentrically before centralizing with growth. In our cohort of 66 children, radial head ossification was eccentric in 68–71% of cases in both sagittal and coronal planes. Offsets were small (average magnitude < 3%), predominantly posterior and radial, and generally did not correlate with age except for progressive centralization in the coronal plane among males. The radiocapitellar line remained reliable on the lateral view but demonstrated expected lateral offset on AP views.

**Summary:**

Eccentric radial head ossification represents a common physiologic pattern that does not reliably centralize with age. Recognition of this variant can reduce misdiagnosis and unnecessary intervention, and has implications for fracture assessment, surgical planning, and longitudinal research. Future studies should include prospective MRI tracking across diverse populations to establish normative ranges and develop quantitative tools for clinical applications.

## Introduction

### Background

The pediatric elbow is a complex and dynamic articulation whose radiographic appearance evolves substantially during growth. It features six secondary ossification centers that appear, enlarge, and ultimately fuse at variable ages, posing significant interpretive challenges even for experienced clinicians [1–4]. The radiocapitellar joint is of particular importance, serving as a key indicator of alignment, deformity, or injury that may warrant intervention. It functions as a marker of skeletal maturity, with the capitellum typically ossifying first around the age of one, followed by the radial head around the age three [5].

As a secondary stabilizer, the radiocapitellar joint plays a central role in elbow stability, forearm rotation, and load transmission, bearing up to 90% of axial load when the elbow is extended [6]. Even minor incongruities can significantly impair the range of motion of forearm supination and pronation [7]. Therefore, a comprehensive understanding of normal radial head development and its relationship to the capitellum is crucial for accurate radiographic interpretation and informed clinical decision-making.

Secondary ossification centers of the elbow are traditionally taught using the CRITOE mnemonic: capitellum, radial head, internal (medial) epicondyle, trochlea, olecranon, and external (lateral) epicondyle (Table 1). Although this sequence remains a valuable educational tool, ossification centers frequently appear asynchronously, display irregular or multifocal morphologies, and fuse in overlapping patterns [8]. Sex differences are well-documented, with ossification occurring earlier in girls compared with boys, typically by one to two years, attributable to accelerated skeletal maturation [9, 10]. These variations are increasingly apparent with advanced imaging modalities such as MRI, which allow for direct visualization of both cartilaginous and ossified components.

**Table 1:** The CRITOE mnemonic often used to remember the typical chronological sequence of ossification centers in the pediatric elbow

Ossification center	Approximate age at initial ossification
**C**apitellum	1
**R**adial head	3
**I**nternal (medial) epicondyle	5
**T**rochlea	7
**O**lecranon	9
**E**xternal (lateral) epicondyle	11

Misinterpretation of normal developmental variants as pathology is a persistent challenge in pediatric elbow imaging. The radial head is especially susceptible to diagnostic errors due to its relatively late ossification, evolving morphology, and proximity to common injury sites, such as radial neck fractures, lateral condyle fractures, and Monteggia equivalents [11, 12]. Furthermore, the radiographic relationship between the radial head and capitellum during development remains poorly defined, with asynchronous and potentially eccentric ossification contributing to normal developmental variability [13, 14]. To assess elbow alignment, the radiocapitellar line (RCL) is commonly used, with deviations often interpreted as signs of fracture or dislocation. However, multiple studies have demonstrated its unreliability in young children, particularly those under five years of age, underscoring the need for a more nuanced understanding of underlying ossification patterns [13, 15, 16].

Morphologic characteristics of these centers vary considerably. The capitellum emerges early as a rounded structure anterior to the distal humerus, often exhibiting initial eccentric ossification before centralizing [13, 14]. It then fuses with the trochlea and lateral epicondyle to form the distal humeral epiphysis. The trochlea may ossify multifocally or appear irregular and sclerotic, potentially mimicking osteochondritis dissecans or a fracture [17]. The olecranon ossification center can present as bipartite, eccentric, or fragmented, especially in adolescents [18]. Epicondylar centers, particularly the medial one, may show irregular margins or delayed fusion, exacerbating diagnostic pitfalls in trauma scenarios. Collectively, these variations highlight the elbow’s developmental complexity, but they are especially impactful at the radiocapitellar joint, where subtle deviations can directly affect fracture diagnosis and management, requiring focused examination of radial head ossification.

## Radial Head Ossification: Normal Patterns

The radial head ossification center typically appears between three and six years of age, positioning it as the second ossification center in the CRITOE sequence [5, 10]. It initially manifests radiographically as a small, rounded density within the cartilaginous epiphysis, and progressively enlarges. Full epiphyseal ossification generally occurs between five and eight years, with fusion to the proximal radial metaphysis (radial neck) between 14 and 17 years, often occurring earlier in girls.

Shape evolution follows a predictable trajectory in anteroposterior (AP) and lateral views. Early stages yield a spherical or ovoid epiphysis centered over the radial neck. As growth proceeds, it expands to a hemispherical contour before flattening superiorly to articulate with the capitellum, concurrent with metaphyseal remodeling that creates a flared “neck” appearance. Metaphyseal modeling changes, such as cortical irregularities or subtle notching, can mimic fractures, particularly near high-risk injury sites like the radial neck. Many such variations are physiologic, emphasizing the importance of age- and sex-specific interpretation to avoid overdiagnosis.

### Eccentric and Atypical Ossification Patterns

Eccentric epiphyseal ossification has been most clearly described in the capitellum by MRI-based studies [13, 14]. These cohort studies demonstrate that capitellar ossification often begins with an anterior and medial offset, followed by progressive centralization during growth. This asymmetry in both the anterior-posterior and medial-lateral planes produces RCL deviations on radiographs, especially in children under five years of age, in whom the line often misses the central third of the capitellum [10, 13, 15, 16]. These observations help explain the limited reliability of the RCL in young patients, and highlights the value of MRI in assessing cartilaginous alignment of the pediatric elbow.

Morphologic variation is not limited to the capitellum. Other secondary ossification centers of the elbow may demonstrate irregular or multifocal ossification, delayed fusion, and variable contour during development. The trochlea may ossify in a fragmented or sclerotic pattern that can mimic osteochondritis dissecans or fracture [17]. The olecranon ossification center may appear as bipartite, eccentric, or fragmented, particularly in adolescents [18]. Similarly, condylar centers, particularly the medial one, can show irregular margins or delayed fusion, exacerbating diagnostic pitfalls in trauma scenarios. Collectively, these variations highlight the elbow’s developmental complexity, but they are especially impactful at the radiocapitellar joint, where subtle deviations can directly affect fracture diagnosis and management, requiring focused examination of radial head ossification.

Although not systematically described for the radial head, parallels with capitellar patterns suggest that similar physiologic variation may occur. Reported variants include multifocal or irregular initial ossification, delayed centralization, and atypical sequences, such as delayed radial head ossification relative to the medial epicondyle [13]. These features may be misinterpreted as pseudo-fractures, apparent dysplasia, or osteochondral lesions, particularly in traumatic contexts such as Monteggia equivalents or lateral condyle fractures [19]. Incomplete or eccentric patterns can also mask subtle radiocapitellar incongruences, contributing to diagnostic errors. To quantify true eccentricity, a reproducible measurement method is essential. In this review, we apply a center-based measurement approach using MRI to objectively evaluate the relationship between the cartilaginous anlage and the ossified nucleus of the radial head in our institutional cohort.

## Institutional Cohort

This retrospective review was conducted at a single academic institution following Institutional Review Board approval. Patients under 18 years of age who underwent upper extremity MRI scans between January 2011 and September 2022 were identified. Inclusion criteria required true coronal and sagittal imaging planes of the elbow, with no evidence of deformity or trauma to the radial head, neck, or capitellum. Indications for imaging varied but primarily included evaluation of pain, suspected pathology (e.g., osteochondritis dissecans), or other non-traumatic elbow concerns; studies with acute trauma or congenital anomalies were excluded to minimize bias toward pathologic variants.

**Fig. 1:**
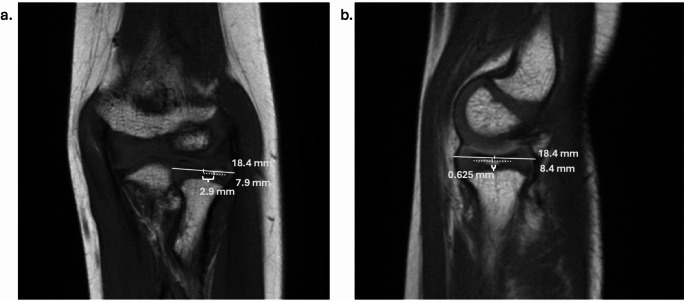
Exemplar measurements of the cartilaginous anlage in the coronal and sagittal planes. (**a**) Coronal view demonstrating measurements of the cartilaginous anlage (18.4 mm), radial head ossific nucleus (7.87 mm), and radial head offset of 3.93 mm radially. (**b**) Sagittal MRI image demonstrating measurements of the cartilaginous anlage (18.4 mm), radial head ossific nucleus (8.44 mm), and anterior (positive) radial head offset of 0.625 mm

**Fig. 2:**
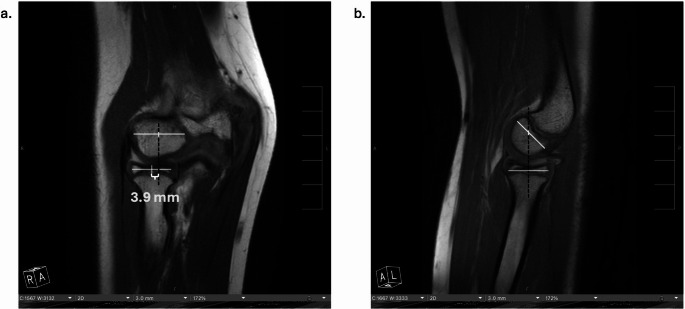
Exemplar measurements of the radiocapitellar offset in the coronal and sagittal planes. (**a**) Coronal MRI demonstrating radial (positive) radiocapitellar offset of 3.9 mm. The maximal medial-lateral dimension of the ossified radial head (dashed lines) and capitellum (solid lines) are shown with hash marks at the true center. (**b**) Sagittal MRI demonstrating no radiocapitellar offset. The maximal anterior-posterior dimension of the ossified radial head (dashed lines) and capitellum (solid lines) are shown with hash marks at the true center

Eccentric radial head ossification was operationally defined as an offset between the centers of the cartilaginous anlage and the ossified nucleus. Offsets were quantified in percentages relative to the maximal dimension of the cartilaginous radial head to normalize for patient size variation. In the coronal plane, radial-ulnar offsets were similarly calculated, with ulnar offsets designated as negative (Fig. 1A). In the sagittal plane, anterior-posterior offsets were measured by dividing the maximal anterior-to-posterior dimension in half to identify the center, and posterior offsets were denoted as negative values (Fig. 1B). Radiocapitellar offsets were also measured on both the sagittal and coronal views (Fig. 2). The center of capitellar ossification was identified in a similar fashion, between the centers of the ossified radial head and capitellum, using a line aligned with the radial head and neck. For fused capitella, the medial border was defined at the intersection of a line parallel to the humeral shaft and the metaphyseal apex. Measurements were performed by a senior orthopedic surgery resident and independently verified by a pediatric musculoskeletal radiologist. Interobserver discrepancies were resolved through discussion and remeasurement to achieve consensus, ensuring high reliability. Descriptive statistics and Pearson correlation analyses were conducted using Microsoft Excel (version 16, 2023) to evaluate associations with age. Results with associated p values < 0.05 was defined as statistically significant.

## Results

The cohort included 66 patients (37 males and 29 females) aged 0–18 years. The mean age was 10.8 ± 4.9 years for males and 9.1 ± 5.3 years for females. At least one measurement was obtained in both the coronal and sagittal planes for each age and sex category, except for coronal measurements in four-year-old females due to inadequate imaging.

**Fig. 3:**
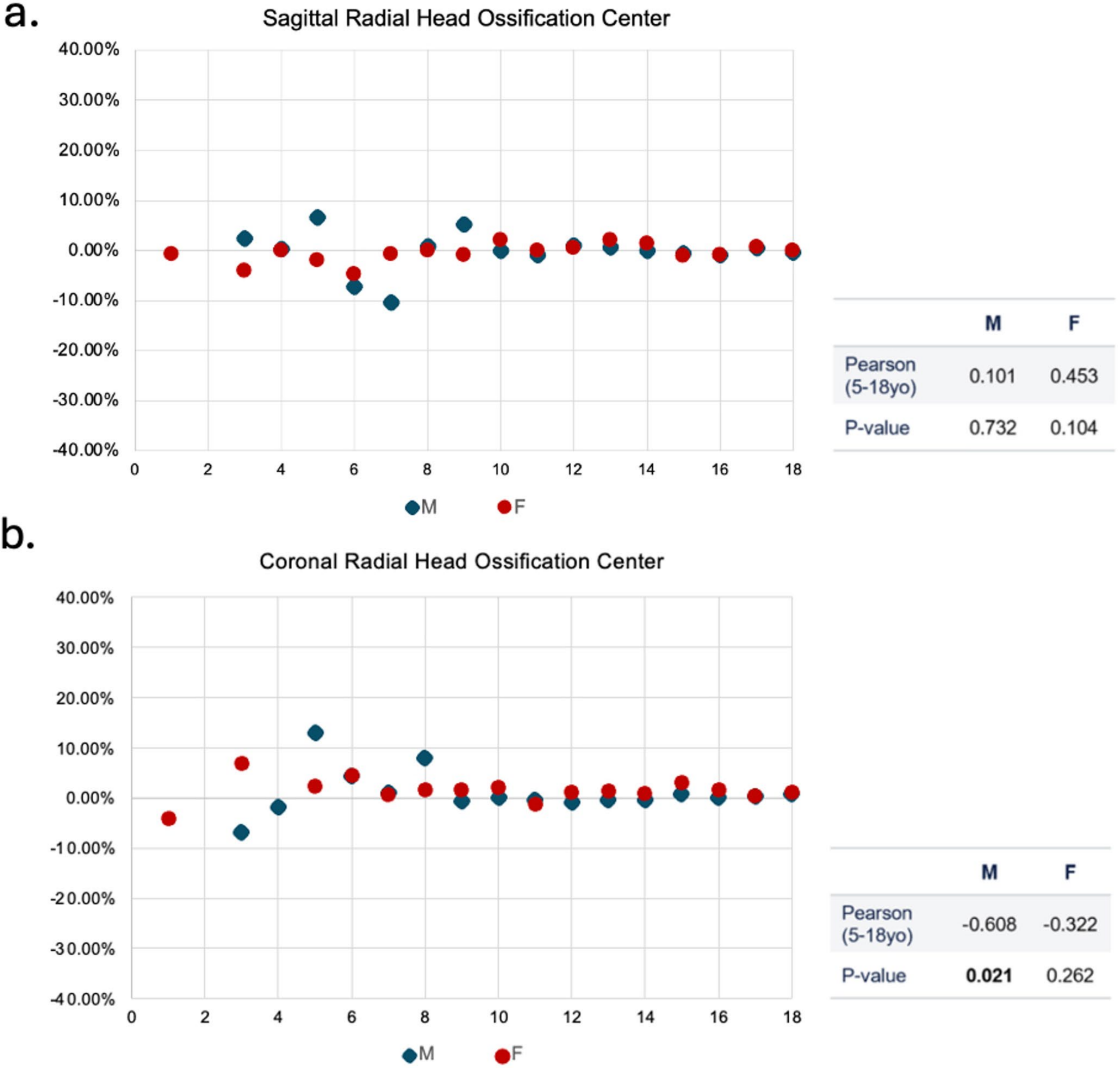
Radial head Ossification Center relative to age amongst males and females (**a**) Radial head ossification center relative to age in the sagittal plane. (**b**) Radial head ossification center relative to age in the coronal plane

In total, 67.6% of radial head ossification centers were eccentric in the sagittal plane, and 71% were eccentric in the coronal plane. There were no significant differences in prevalence by sex (males: 68% sagittal, 70% coronal; females: 67% sagittal, 72% coronal). Among patients with eccentric ossification centers, the offset was more frequently posterior and radial in direction. However, the magnitude of offset was small. In the sagittal plane, the average anterior offset was 2.7%, and the average posterior offset was − 2.8%. In the coronal plane, the average radial offset was 2.8%, and the average ulnar offset was − 2.4%. There was no statistically significant correlation between radial head ossification center location and age in the sagittal plane (males: Pearson coefficient 0.10, *p* = 0.73; females: 0.45, *p* = 0.10) (Fig. 3A). In the coronal plane, a statistically significant correlation was found between decreasing radial head ossification center offset and increasing age in males (Pearson coefficient − 0.61, *p* = 0.02) but not in females (-0.32, *p* = 0.26) (Fig. 3B).

**Fig. 4:**
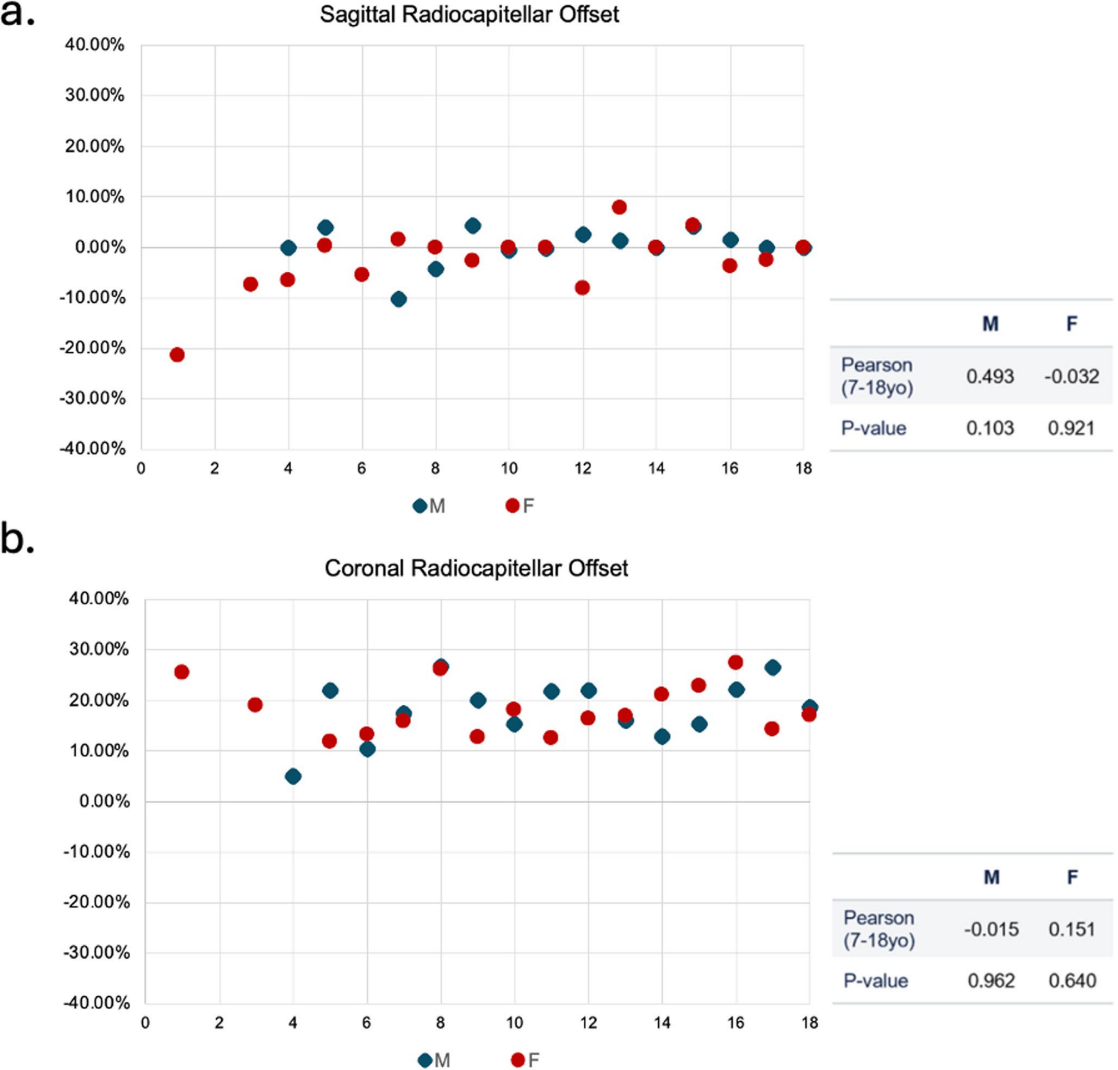
Radiocapitellar offset relative to age amongst males and females. (**a**) Radiocapitellar offset relative to age in the sagittal plane. (**b**) Radiocapitellar offset relative to age in the coronal plane

Radiocapitellar offset was determined for all patients. The magnitude of radiocapitellar offset in the sagittal plane across all ages was, on average, 0.2% for males and − 2.6% for females (Fig. 4A). In the coronal plane, the average radiocapitellar offset was larger, 18% in both males and females (Fig. 4B). There was no significant correlation between the magnitude of offset and age in the sagittal plane (males: Pearson coefficient 0.49, *p* = 0.10; females − 0.03, *p* = 0.92) or in the coronal plane (males: -0.015, *p* = 0.96; females: 0.15, *p* = 0.64).

## Discussion

### Institutional Takeaways

The observed eccentric radial head ossification patterns contrast with those of the capitellum, where eccentricity is more pronounced (10–18% offsets) and typically occurs in an anterior-medial direction, decreasing with age in both sexes. In capitellar studies, such as those by Fader et al., offsets centralize progressively, aligning with overall elbow ossification timelines where the capitellum ossifies earliest (around age 1) and fuses by 10–14 years [13, 14]. Radial head patterns, however, show smaller offsets (< 3% on average), with a posterior-radial predominance, and sex-specific temporal changes (centralization in males only), with no overall age correlation.

Radiocapitellar offset in the coronal plane averaged 18% for both males and females. This is consistent with Fader et al. 2016’s findings, which demonstrated that the radiocapitellar offset, when present, exclusively deviates laterally [13]. Fader et al. attributed this to the medial pattern of capitellar ossification. In the context with our study results, however, lateral radiocapitellar offset appears to be due to eccentric ossification of both the radial head and the capitellum. Clinically, lateral radiography may be more helpful in confirming radiocapitellar alignment and a degree of offset can be expected on the anterior-posterior view.

Our findings support eccentric radial head ossification as a physiologic variation rather than a pathologic marker. The lack of correlation with age, symptoms, or injuries, coupled with persistence into adolescence, mirrors capitellar eccentricity, and could explain limited reliability of the RCL in young children. The high prevalence of eccentricity in both sagittal and coronal planes, the small magnitude of offset, and the lack of consistent correlation with age collectively suggest that mild asymmetry in ossification should be anticipated during development. Importantly, eccentricity persisted into later adolescence, particularly in the coronal plane, reinforcing that incomplete centralization should not be assumed to represent abnormal alignment. These observations may have several practical implications for imaging interpretation and clinical management.

### Distinguishing Physiologic Eccentricity from Pathology

Accurate differentiation between physiologic eccentric ossification and true pathology is critical in pediatric elbow evaluation. In normal eccentric ossification, the cartilaginous anlage remains smooth and continuous, without focal disruption, collapse, or marrow edema. Although the ossified nucleus may appear offset within the cartilaginous epiphysis, overall articular congruity is preserved. In contrast, fractures typically demonstrate cortical discontinuity, marrow edema, or periosteal reaction on MRI, findings that are absent in isolated developmental asymmetry. Extrapolating from our institutional data, the magnitude of offset in the sagittal plane was small compared with that observed in the coronal plane. These findings suggest that the radiocapitellar line remains a useful measure for alignment on the lateral view, although it should be interpreted with greater caution on the anteroposterior view, where a degree of lateral offset may be physiologic.

In our cohort, average offsets were less than 3% in both sagittal and coronal planes, supporting the interpretation that small degrees of asymmetry likely fall within physiologic developmental variation. Larger deviations, particularly when accompanied by cortical irregularity, edema, or persistent clinical symptoms, may warrant further evaluation. These distinctions are especially important in younger children, in whom incomplete ossification already complicates radiographic interpretation. Recognition of physiologic eccentricity may add to existing body of work describing variability in the radiocapitellar line in young children.

### Implications for Trauma Evaluation and Surgical Decision-Making

Understanding normal radial head ossification patterns has direct implications for fracture assessment. Radial neck fractures, Monteggia equivalents, and lateral condyle injuries frequently rely on radiocapitellar alignment for diagnosis and classification. When the ossified radial head is eccentrically positioned within the cartilage, apparent displacement on radiographs may not necessarily reflect true articular incongruity. In such cases, MRI can help determine whether perceived malalignment represents true radiocapitellar instability or physiologic developmental asymmetry.

Although our data does not establish a causal relationship between eccentric ossification and fracture patterns, they suggest that reliance on ossified landmarks alone may be insufficient in skeletally immature patients. Similarly, surgical planning based solely on apparent radiographic incongruity should consider developmental variation. Mild offset in the absence of cartilage disruption or instability may not in itself justify operative intervention,

### Reporting Considerations

Radiology reports may benefit from explicitly acknowledging normal developmental variability. When eccentric ossification is present without edema, displacement, or cortical disruption, describing the finding as a “developmental variant” or “physiologic asymmetry” may reduce diagnostic ambiguity and limit unnecessary immobilization or additional imaging. Orthopaedic documentation should likewise incorporate age- and sex-specific developmental expectations.

Given the persistence of coronal plane radiocapitellar offset without clear age correlation in our cohort, minor deviations should not automatically be interpreted as malalignment. The radiocapitellar line remains a useful screening tool for gross dislocation, but its limitations in young children and in the setting of eccentric ossification must be recognized. Careful assessment of the lateral view is particularly important, as even small sagittal offsets may influence radiographic interpretation.

### Future Directions

Our institutional data is limited by its retrospective, cross-sectional design. Although offsets were quantified across a range of ages, longitudinal imaging of individual patients would better clarify whether true centralization occurs over time and whether patterns differ by sex. Prospective cohort studies tracking radial head ossification from early childhood through skeletal maturity may better provide insight into temporal evolution. Advanced imaging techniques, including three-dimensional MRI reconstruction and emerging sequences capable of depicting cortical bone signal (such as ultrashort or zero-echo-time imaging), may further refine understanding of spatial relationships between cartilage and ossification centers. Additionally, correlating ossification patterns with biomechanical modeling approaches could help determine whether eccentricity has functional consequences. Finally, multicenter studies across diverse populations may clarify potential ethnic or demographic differences in ossification patterns, as suggested in prior studies on elbow development.

## Conclusions

In summary, we found that radial head ossification demonstrates physiologic variability that is not fully captured by traditional teaching frameworks such as CRITOE. In our cohort, eccentric ossification was present in approximately two-thirds of patients in both sagittal and coronal planes. Offsets were small in magnitude, most frequently posterior and radial, showed little correlation with age, and commonly persisted into adolescence, while sagittal radiocapitellar congruity remained preserved. These patterns parallel, yet remain distinct from, the more pronounced anterior-medial eccentricity described in the capitellum and may contribute to the previously described limitations of the radiocapitellar line in young children.

Together, these findings support a more developmentally informed, context-driven approach to pediatric elbow imaging. Clinically, mild asymmetry on radiographs should not be overinterpreted in the absence of findings suggestive of true injury such as cortical disruption, instability, or persistent clinical symptoms. In cases where radiographic findings are equivocal, MRI may serve as a useful adjunct to assess the cartilaginous anlage and confirm articular congruity. These considerations are particularly relevant in younger patients in whom incomplete ossification already complicates imaging assessment. Establishing normative ranges through larger, prospective, and longitudinal studies will be essential to refine diagnostic thresholds and further guide evidence-based interpretation of the developing pediatric elbow.

## Key References

- Fader LM, Laor T, Eismann EA, Cornwall R, Little KJ. MR imaging of capitellar ossification: a study in children of different ages. Pediatr Radiol. 2014;44:963–70. 10.1007/s00247-014-2921-4
  Using MRI, this foundational work maps the eccentric, age-dependent pattern of capitellar ossification. The quantitative analysis of ossific nucleus position relative to the cartilaginous anlage was the blueprint closely for our center-based offset measurements for the radial head.
- Fader LM, Laor T, Eismann EA, Cornwall R, Little KJ. Eccentric Capitellar Ossification Limits the Utility of the Radiocapitellar Line in Young Children. Journal of Pediatric Orthopaedics. 2016;36:161–6.10.1097/BPO.0000000000000426
  This pivotal study systematically demonstrates that eccentric ossification of the capitellum is a common physiologic phenomenon in young children, frequently producing apparent deviations of the radiocapitellar line (RCL) on radiographs that do not reflect true joint malalignment. By correlating radiographic findings with clinical context, the authors establish that the RCL intersects the central third of the capitellum less reliably in skeletally immature elbows, particularly on anteroposterior views.
- Kim AE, Chi H, Kammen B, Livingston K, Zapala M, Swarup I. Utility of Fast MRIs in Pediatric Elbow Injuries. Journal of the Pediatric Orthopaedic Society of North America. 2024;7:100026.10.1016/j.jposna.2024.100026
  Recent study highlighting the practical value of novel “fast” MRI in evaluating pediatric elbow trauma when radiographs are equivocal. They demonstrate how fast MRI clarifies alignment without the time or sedation burdens of conventional scans, and its use as an adjunct in making clinical decisions when radiography is unclear. These findings align with our recommendations for using MRI to distinguish physiologic radial head offsets from true articular disruption, while offering institutional guidance on protocol implementation.
- Kashayi-Chowdojirao S, Yadala DR. Pediatric Elbow – Developmental and Radiological Anatomy. Journal of Orthopaedic Association of South Indian States. 2022;19(Suppl 1):S9–18.10.4103/2667-3665.346027
  This is a recent detailed recent synthesizing the sequence and morphologic evolution of the elbow’s secondary ossification centers, complete with radiographic landmarks and a gallery of normal variants that aid differentiation from pathologic appearances. It addresses asynchronous development, irregular contours, and sex-based timing differences, providing a broad anatomic foundation for understanding why eccentric patterns at the radiocapitellar joint are frequently misinterpreted.
- Lee J, Cha Y, Kang MS, Park SS. Growth of the Capitellar Ossification Center and Its Relationship within the Lateral Condyle of the Distal Humerus in Skeletally Immature Elbows: A Study Using MR Images. Journal of Pediatric Orthopaedics B. 2020;29(2):187–94.10.1097/BPB.0000000000000673
  Employing a larger MRI cohort and a similar quantitative approach to ours and the Fader studies, this more recent investigation adds to the literature on the capitellar eccentric growth. The findings corroborate the Fader et al. studies in regard to age-related changes in ossification center position and their impact on standard radiographic lines.

## Data Availability

Data supporting the findings of this study are available within the paper. Raw data generated in this study is also available upon request.
